# Intracerebral Administration of Recombinant Rabies Virus Expressing GM-CSF Prevents the Development of Rabies after Infection with Street Virus

**DOI:** 10.1371/journal.pone.0025414

**Published:** 2011-09-28

**Authors:** Hualei Wang, Guoqing Zhang, Yongjun Wen, Songtao Yang, Xianzhu Xia, Zhen F. Fu

**Affiliations:** 1 Department of Pathology, University of Georgia, Athens, Georgia, United States of America; 2 Institute of Veterinary Medicine, Chinese Academy of Military Medical Sciences, Changchun, Jilin, China; 3 College of Animal Science and Veterinary Medicine, Jilin University, Changchun, Jilin, China; 4 Department of Infectious Diseases, University of Georgia, Athens, Georgia, United States of America; 5 State Key Laboratory of Agricultural Microbiology, Huazhong Agricultural University, Wuhan, China; Indian Institute of Science, India

## Abstract

Recently it was found that prior immunization with recombinant rabies virus (RABV) expressing granulocyte-macrophage colony-stimulating factor (GM-CSF) (LBNSE-GM-CSF) resulted in high innate/adaptive immune responses and protection against challenge with virulent RABV (Wen et al., JVI, 2011). In this study, the ability of LBNSE-GM-CSF to prevent animals from developing rabies was investigated in mice after infection with lethal doses of street RABV. It was found that intracerebral administration of LBNSE-GM-CSF protected more mice from developing rabies than sham-treated mice as late as day 5 after infection with street RABV. Intracerebral administration of LBNSE-GM-CSF resulted in significantly higher levels of chemokine/cytokine expression and more infiltration of inflammatory and immune cells into the central nervous system (CNS) than sham-administration or administration with UV-inactivated LBNSE-GM-CSF. Enhancement of blood-brain barrier (BBB) permeability and increases in virus neutralizing antibodies (VNA) were also observed in mice treated with LBNSE-GM-CSF. On the other hand, intracerebral administration with UV-inactivated LBNSE-GM-CSF did not increase protection despite the fact that VNA were induced in the periphery. However, intracerebral administration with chemoattractant protein-1 (MCP-1, also termed CCL2) increased significantly the protective efficacy of UV-inactivated LBNSE-GM-CSF. Together these studies confirm that direct administration of LBNSE-GM-CSF can enhance the innate and adaptive immunity as well as the BBB permeability, thus allowing infiltration of inflammatory cells and other immune effectors enter into the CNS to clear the virus and prevent the development of rabies.

## Introduction

Rabies is one of the oldest zoonotic diseases and today it continues to present a serious burden to the public health and the global economy. It causes more than 55,000 human deaths and more than 15 million people undergo post-exposure prophylaxis (PEP) every year around the globe 1], which is responsible for 1.74 million disability adjusted life year score (DALYs) lost 2]. Most of the human cases occur in the developing countries of Asia and Africa where canine rabies is endemic and resources are limited 3]. In more developed countries, human rabies has dramatically declined during the past 60 years as a direct consequence of routine vaccination of pet animals 4]. However, wildlife rabies has emerged as a major threat. In the United States, more than 90% of animal rabies cases have been reported in wildlife such as raccoons, bats, skunks and foxes 5,6]. Although there have been incidents in which large carnivores transmit rabies directly to humans 7,8], most of the human cases (>90%) in the past two decades are associated with rabies virus (RABV) found in bats, particularly the silver-haired bats (SHBRV) 8,9,10]. Unlike classical rabies, which is inflicted by the bite of an infected animal, these newly emerging human rabies infections occurred without a known history of exposure 8]. As a result, PEP with vaccines and anti-rabies immunoglobulin could not be initiated. This combined treatment is effective when it is initiated within a few days (but as soon as possible) 11]. However, delayed treatment with rabies vaccines currently in use may actually accelerate the development of rabies 12]. It is widely accepted that there is no proven effective treatment and rabies is almost invariably fatal once clinical symptoms of rabies develop 12]. Thus new modalities are needed to prevent and treat clinical rabies.

Recently it was found that activation of the innate immunity, particularly type I interferon (IFN) and chemokines, is one of the mechanisms by which RABV is attenuated 13]. Furthermore, infection with rRABV expressing chemokines/cytokines resulted in further attenuation of viral virulence and enhancement of innate and adaptive immunity by inducing the expression of innate immune molecules, the infiltration of inflammatory cells into the CNS, and the enhancement of BBB permeability 14]. For example, prior immunization with rRABV expressing chemokines/cytokines MDC (macrophage-derived chemokine) or GM-CSF (granulocyte-macrophage colony-stimulating factor) induced significantly more VNA responses in mice, which led to better protection than with the parent virus when challenged with virulent RABV 15,16]. Yet, these rRABVs did not induce any overt disease or death in adult mice when 10^7^ fluorescent focus unit (FFU) was directly inoculated by the intracerebral (ic) route 16]. In the present study, rRABV expressing GM-CSF (LBNSE-GM-CSF) was used to treat mice at different days after an intramuscular infection with a lethal dose of street RABV. It was found that direct intracerebral administration of LBNSE-GM-CSF could induce innate and adaptive immune responses, allowing immune effectors enter into the CNS to clear the virus and prevent the development of rabies as late as 5 days after infection.

## Results

### Treatment with LBNSE-GM-CSF can prevent mice from developing rabies as late as 5 days after infection with street RABV

**Figure 1:**
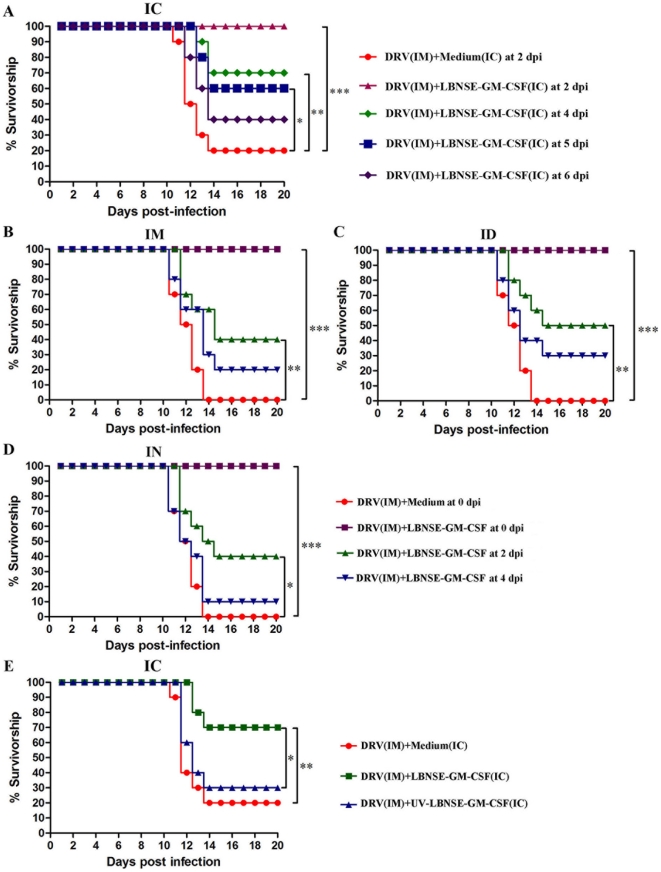
Protective efficacy of recombinant RABVs administrated after infection i.m. with DRV. ICR mice (group of 10) at age of 4–6 weeks were infected i.m. with 10 IMLD_50_ DRV, and treated with 10^7^ FFU LBNSE-GM-CSF by intracerebral (A), intramuscular (B), intradermal (C) or intranasal (D) route at different time point post infection, or treated with live or UV-inactivated LBNSE-GM-CSF at day 4 post infection (E). Infected and treated mice were observed daily for 20 days and survivorship was recorded and analyzed. Asterisk indicates significant differences between the indicated experimental groups as calculated by Log-rank test: *, p<0.05; **, *p<0.01;* ***, *p<0.001.*

Our previous studies indicated that rRABVs expressing chemokines/cytokines, e.g. GM-CSF or MDC, activate/recruit DCs and enhance protective immune responses when given before challenge 16]. To investigate whether these rRABVs could prevent mice from developing rabies when administered after challenge, ICR mice were inoculated by intramuscular (im) route with 10 50% intramuscular mouse lethal dose (IMLD_50_) of street RABV (DRV) and then treated with 10^7^ FFU of LBNSE-GM-CSF by ic route at 2, 4, 5, and 6 days post infection (dpi). Mice were monitored daily for 20 days for developing disease and death. By dpi 6 to 8, animals began to develop clinical sign such as ruffled fur; trembling and shaking; incoordinated movement; and paralysis. Mice were humanely sacrificed when developing complete paralysis by dpi 10 to 13. As shown in Fig. 1A, 80% of the mice infected with DRV by im, but treated with medium by ic at 2 dpi developed rabies and succumbed to infection by 14 dpi. None of DRV-infected mice developed any clinical symptom when treated by ic with LBNSE-GM-CSF at 2 dpi. Significantly more mice (70%) survived the infection when treated with LBNSE-GM-CSF at 4 dpi (*p<0.01*). When the treatment was initiated at 5 dpi, 60% of the mice survived and this is significant when compared to shamed-treated. When the treatment was initiated at 6 dpi, 40% of the mice were protected, but no significant difference was found when compared with sham-treated mice. These data indicate that LBNSE-GM-CSF can prevent mice from developing rabies when administered ic as late as 5 dpi. To further investigate if ic administration of UV-inactivated LBNSE-GM-CSF has the same ability to prevent animals from developing rabies, mice were infected with DRV and treated with the same amount (equivalent to 10^7^ FFU) of UV-inactivated LBNSE-GM-CSF at 4 dpi. (Fig. 1E). No significantly more protection was observed in mice treated with UV-inactivated LBNSE-GM-CSF than in sham-treated mice, indicating that treatment with UV-inactivated virus is not able to prevent rabies in the mouse model.

To address if the LBNSE-GM-CSF is also efficacious when administered by other routes, mice were infected by im with 10 IMLD_50_ DRV and treated with 10^7^ FFU LBNSE-GM-CSF at 0, 2, or 4 dpi by im (Fig. 1B), intradermal (Fig. 1C) or intranasal (Fig. 1D) route. All the infected and sham-treated mice died while no mortality was observed in mice when LBNSE-GM-CSF was administrated at 0 dpi by each route. Significantly more protection (*p<0.01* or *p<0.05*) was observed when mice were treated at 2 dpi (40–50%). No significant protection was observed when treatment was initiated by 4 dpi, indicating that peripheral route is not as effective as the ic route of administration for treatment with LBNSE-GM-CSF.

### Treatment with LBNSE-GM-CSF cleared RABV from the CNS

To investigate if treatment with LBNSE-GM-CSF leads to virus clearance from the CNS, DRV-infected mice were treated ic at 4 dpi with 10^7^ FFU LBNSE-GM-CSF or UV-inactivated LBNSE-GM-CSF (equivalent to 10^7^ FFU). Brains were harvested and virus titers determined at days 3, 6, 9 after treatment (Table 1). Higher virus titers were detected in the sham-treated mice or mice treated with UV-inactivated LBNSE-GM-CSF at day 6 and 9 post treatment. In LBNSE-GM-CSF-treated group, virus was detected in all three mice at day 3, none at day 6, and only one mouse at day 9 post treatment (with low titer). These data indicate that treatment with LBNSE-GM-CSF not only results in clearance of the recombinant virus, but also street RABV from the CNS.

**Table 1:** Virus titers in the brain of mice infected im with DRV (10 IMLD_50_) and treated with rRABVs at 4 dpi.

Treatment	Medium	rRABV (GM-CSF) *	UV-inactivated rRABV (GM-CSF)*
D3	-†, -, -	10^2.5^, 10^2^, 10^2.5^	-, -, -
D6	10^4.5^, 10^3.5^, 10^5^	-, -, -	10^4^, 10^3.5^, 10^4^
D9	10^5^	10^3^, -, -	10^5^, 10^3^, 10^4^

*rRABV (GM-CSF), recombinant RABV expressing GM-CSF.

† Virus titer in the brain (FFU/g tissue).

- No virus was detected.

### Treatment with LBNSE-GM-CSF induced expression of chemokines and cytokines in the brain

**Figure 2:**
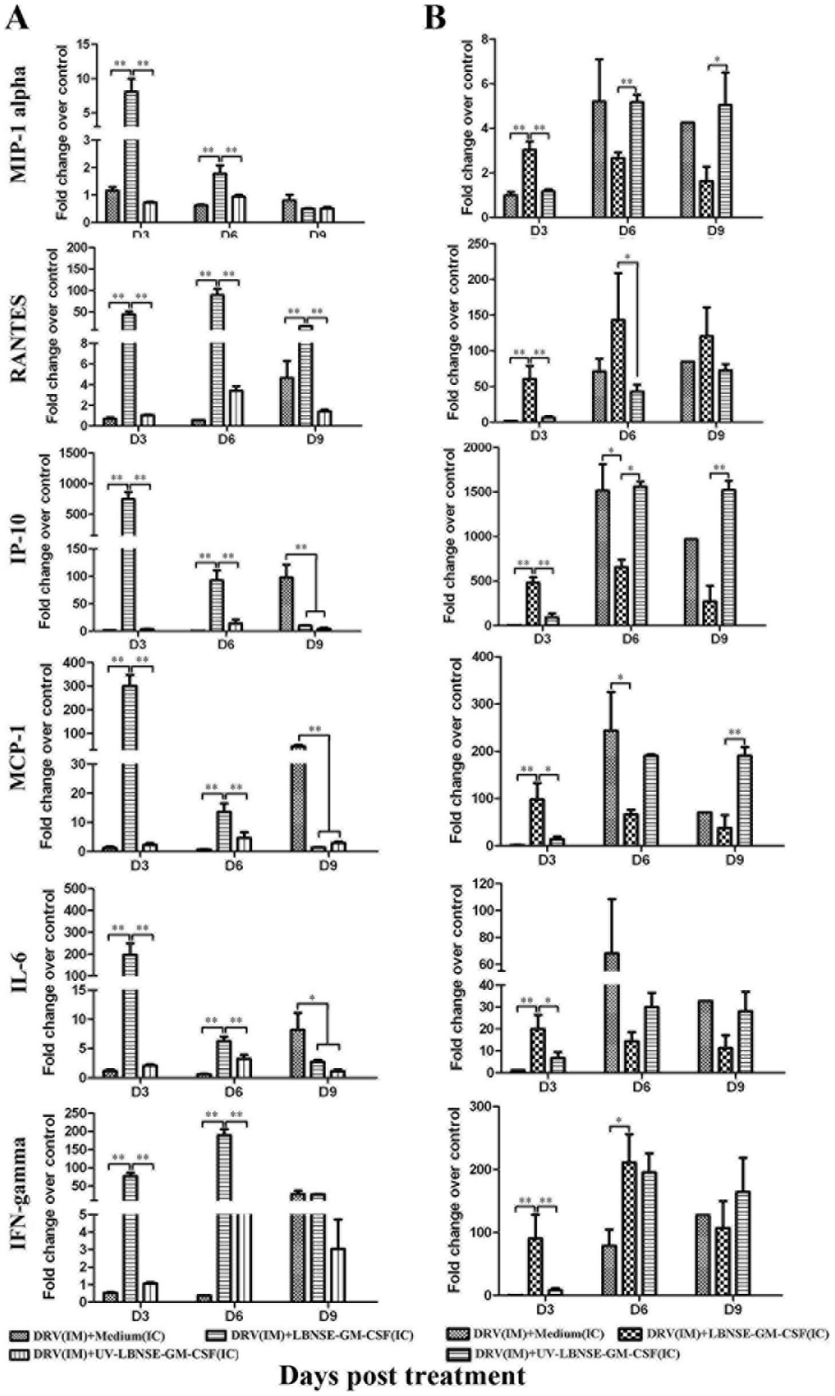
Concentration of chemokines and cytokines in the brain of mice treated with LBNSE-GM-CSF. BALB/c mice were infected with 10 IMLD_50_ DRV and treated at either 0 (A) or 4 (B) dpi with medium, 10^7^ FFU live or UV-inactivated LBNSE-GM-CSF, brain samples were collected at days 3, 6, and 9 post treatment. Total RNA was extracted from the brain tissue and mRNA of chemokines and cytokines was analyzed by qRT-PCR. The mRNA copy number was normalized to the housekeeping gene GAPDH. Levels of gene expression in a test sample are presented as the fold change over that detected in sham-infected controls. Data represent the average from three independent experiments. Asterisk indicates significant differences between the indicated experimental groups as calculated by one-way ANOVA: *, p<0.05; **, *p<0.01.*

To determine the mechanism(s) by which treatment with LBNSE-GM-CSF clears RABV from the brain, the expression of chemokine and cytokine (MIP-1 alpha, RANTES, IP-10, MCP-1, IL-6, and IFN-gamma) mRNA was measured by qRT-PCR in the brain. In mice infected with DRV and treated with LBNSE-GM-CSF at the same time (Fig. 2A), the expression level of all these chemokines/cytokines was highly up-regulated at day 3 post treatment. By day 6 post treatment, the expression level for most of these chemokines/cytokines was reduced drastically although remained significantly higher than in mice sham-treated or treated with UV-inactivated LBNSE-GM-CSF. However, the level of RANTES expression remained elevated and the level of IFN-gamma expression reached a peak by day 6 post treatment. By day 9 post treatment, only RANTES remains at high level in mice infected with DRV and treated with LBNSE-GM-CSF. On the other hand, high levels of IP-10, MCP-1, and IL-6 were detected in mice infected with DRV, but sham-treated, by day 9 post treatment.

When the treatment was initiated at 4 dpi, the expression level of chemokines/cytokines was significantly higher in the brain of mice treated with LBNSE-GM-CSF than in mice sham-treated or treated with UV-inactivated LBNSE-GM-CSF (Fig. 2B) at day 3 post treatment. By days 6 and 9 post treatment (10 and 13 dpi), however, significantly higher levels of chemokines, particularly MIP-1 alpha, IP-10 and MCP-1 were detected in the brain of mice sham-treated or treated with UV-inactivated LBNSE-GM-CSF than in mice treated with live LBNSE-GM-CSF. For RANTES and IFN-gamma, the level was significantly higher in mice treated with LBNSE-GM-CSF at day 6, but not at day 9 post treatment.

Overall, these data indicate that direct ic administration of LBNSE-GM-CSF is capable of inducing high and transient expression of chemokines/cytokines in the CNS at day 3 post treatment (early innate immune responses). On the other hand, DRV only is capable of inducing expression of chemokine/cytokine at late stage of infection (9 dpi or later). Direct ic administration of UV-inactivated LBNSE-GM-CSF does not induce chemokine/cytokine expression.

### Treatment with LBNSE-GM-CSF induced infiltration of inflammatory cells into the mouse brain

**Figure 3:**
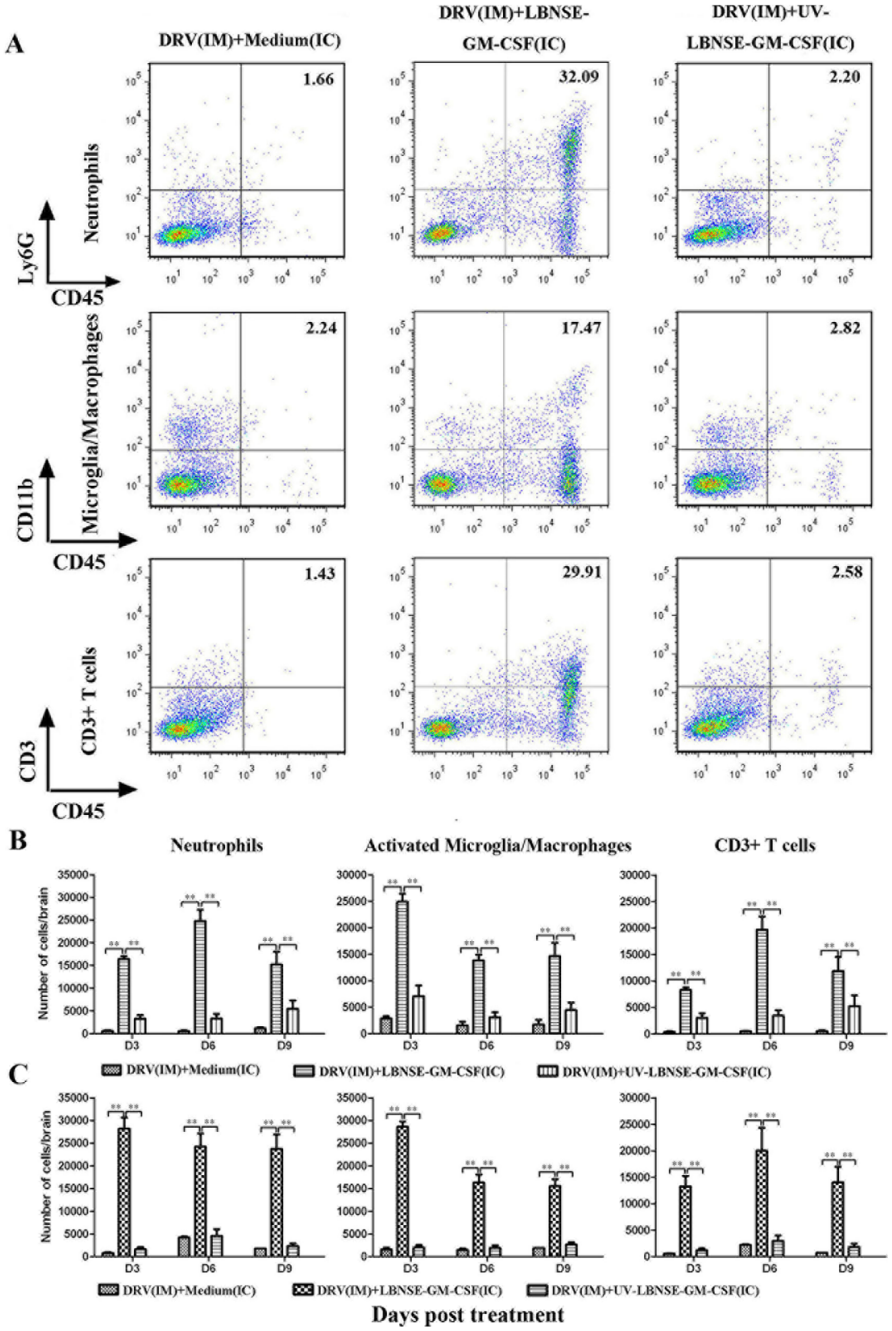
Differentiation of inflammatory cells infiltrated into CNS by flow cytomeric analyses. BALB/c mice were infected i.m. with 10 IMLD_50_ DRV and were treated at either 0 or 4 dpi with medium, 10^7^ FFU live or inactivated LBNSE-GM-CSF. Leukocytes from CNS were recovered by Percoll centrifugation and stained with cell surface markers CD11b (Microglia/macrophage), Ly6G (Neutrophils) and CD3 (T cells). The stained cells were analyzed by flow cytometry. Representative flow cytometric plots of infiltrated cells in mouse brain at day 6 post treatment for each of the treatment groups (A). The absolute numbers of indicated inflammatory cells in the brain were presented for mice treated at day 0 (B) or 4 (C) dpi, respectively. All data are from n = 3 mice per group. Asterisk indicates significant differences between the indicated experimental groups as calculated by one-way ANOVA: *, p<0.05; **, p<0.01.

To investigate if expression of chemokins/cytokines leads to infiltration of inflammatory cells into the CNS, flow cytometry was performed to analyze the leukocytes infiltrated into the mouse brain at days 3, 6, 9 post treatment. Fig. 3A shows representative flow cytometric plots of neutrophils (Ly6G+ and CD45+), activated microglia/macrophages (CD11b+ and CD45+) and CD3+ T cells (CD3+ and CD45+) from brain of mice infected with DRV and treated with LBNSE-GM-CSF. Detailed data are presented in Fig. 3B for mice infected with DRV and treated at the same time and in Fig. 3C for mice infected with DRV and treated at 4 dpi. Significantly more infiltration of activated microglia/macrophages (*p<0.01*), neutrophils (*p<0.01*) and CD3+ T cells (*p<0.01*) was observed in mice infected with DRV and treated with LBNSE-GM-CSF than in mice infected with DRV and sham-treated or treated with UV-inactivated LBNSE-GM-CSF at days 3, 6, 9 post treatment (Fig. 3B, 3C). These data indicate that inflammatory cells were trafficking to and accumulating in the CNS of mice treated with live LBNSE-GM-CSF.

### Treatment with LBNSE-GM-CSF resulted in the enhancement of BBB permeability

**Figure 4:**
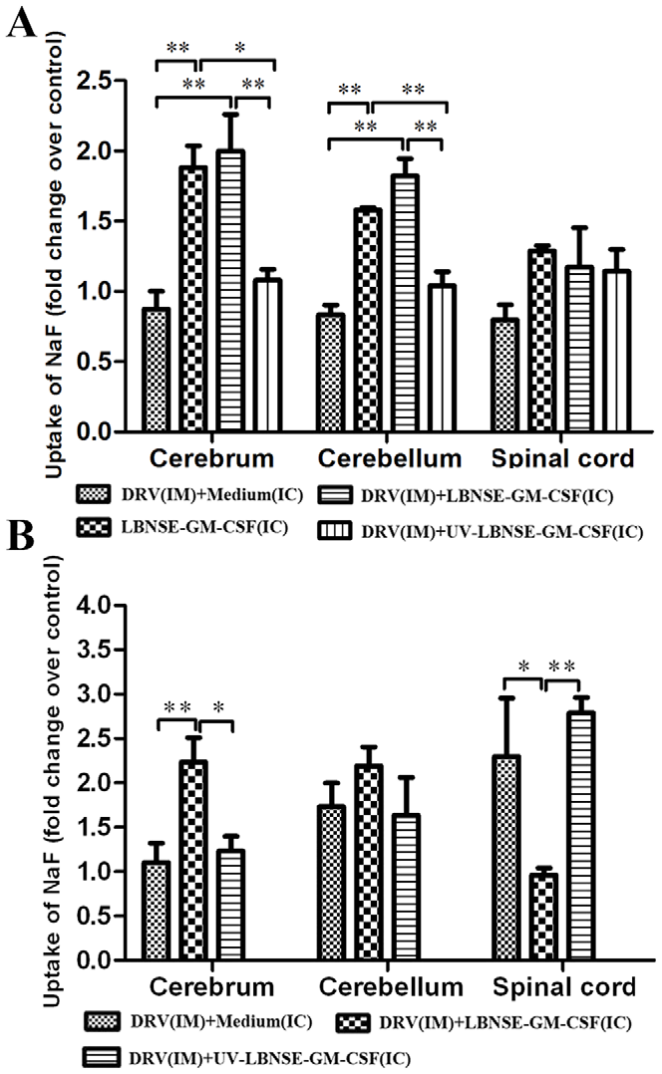
Induction of BBB permeability after treatment with LBNSE-GM-CSF. Mice were treated with medium or 10^7^ FFU LBNSE-GM-CSF at 0 (A) or 4 (B) dpi. BBB permeability was determined by uptake of sodium fluorescein at day 6 post treatment. Data are given as mean values + SEM. Asterisks indicate significant differences between the indicated experimental groups: *, p<0.05; **, p<0.01.

Previous studies have demonstrated that enhancement of BBB permeability is one of the important mechanisms by which the immune effectors can enter into the CNS to clear RABV 17,18,19]. To investigate if treatment with LBNSE-GM-CSF enhances BBB permeability, the leakage of sodium fluorescein (NaF) from the circulation into CNS tissues was measured in the cerebrum, cerebellum or spinal cord at day 6 post treatment. As shown in Fig. 4A, BBB permeability to NaF was significantly elevated in the cerebrum and cerebellum of mice treated with LBNSE-GM-CSF with or without prior infection with DRV when compared with that in sham-treated mice or in mice treated with UV-inactivated LBNSE-GM-CSF. When the treatment was initiated 4 dpi, significantly more increase in BBB permeability was detected in the cerebrum of mice treated with LBNSE-GM-CSF than in mice sham-treated or treated with UV-inactivated LBNSE-GM-CSF (Fig. 4B). On the other hand, BBB permeability of mice infected with DRV without treatments or treated with UV-inactivated LBNSE-GM-CSF was enhanced. These data indicate that indeed treatment with LBNSE-GM-CSF enhances the BBB permeability in the CNS (cerebrum and cerebellum) at day 6 post treatment. Street RABV alone is only capable of enhancing the BBB permeability in the spinal cord at late stage of infection (10 dpi).

### Treatment with LBNSE-GM-CSF resulted in recruitment/activation of DCs and B cells

**Figure 5:**
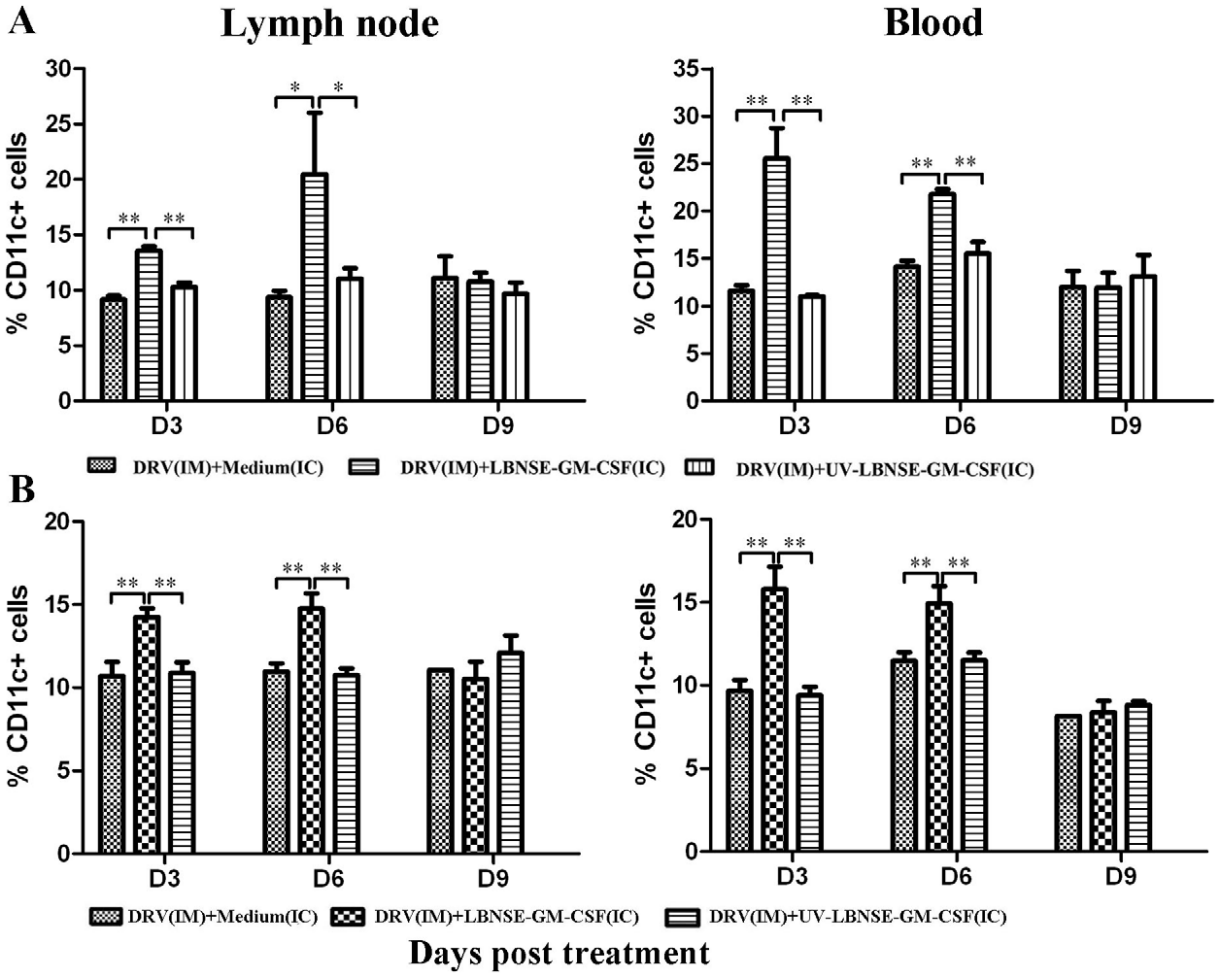
Recruitment/activation of DCs in the lymph node and periphery blood of mice treated with LBNSE-GM-CSF. BALB/c mice were infected with 10 IMLD_50_ DRV and treated at 0 (A) or 4 (B) dpi with medium, 10^7^ FFU live or inactivated LBNSE-GM-CSF. At days 3, 6, and 9 post treatment, single cell suspensions were prepared from inguinal lymph node or peripheral blood and stained with antibodies against surface markers of DCs (CD11c+). The stained cells were analyzed by flow cytometry. All data are from n = 3 mice in each group and presented as mean values ± standard errors (SE). Asterisk indicates significant differences between the indicated experimental groups as calculated by one-way ANOVA: *, p<0.05; **, p<0.01.

To investigate if stimulation of innate immunity (expression of chemokines/cytokines and infiltration of inflammatory cells) enhances the adaptive immunity, the recruitment/activation of DCs and B cells was analyzed by flow cytometry using antibodies to cell surface markers (CD11c, CD86 and CD80 for activated DCs, CD19 and CD40 for B cells). Fig. 5 shows recruitment/activation of DC in mice infected and treated at the same time (Fig. 5A) or treated at 4 dpi (Fig. 5B). Significantly more DCs (CD11c+) were detected at days 3 and 6 post treatment in mice treated with LBNSE-GM-CSF than in sham-treated mice or mice treated with UV-inactivated LBNSE-GM-CSF. Expression level of activated DC markers (CD80 and CD86) was significantly up-regulated at days 3 and 6 post treatment in mice treated with LBNSE-GM-CSF than in sham-treated mice or in mice treated with UV-inactivated virus as shown by the geometric mean fluorescence (data not shown). By day 9 post treatment, the activation of DC subsided and no significant difference was found among the different groups.

**Figure 6:**
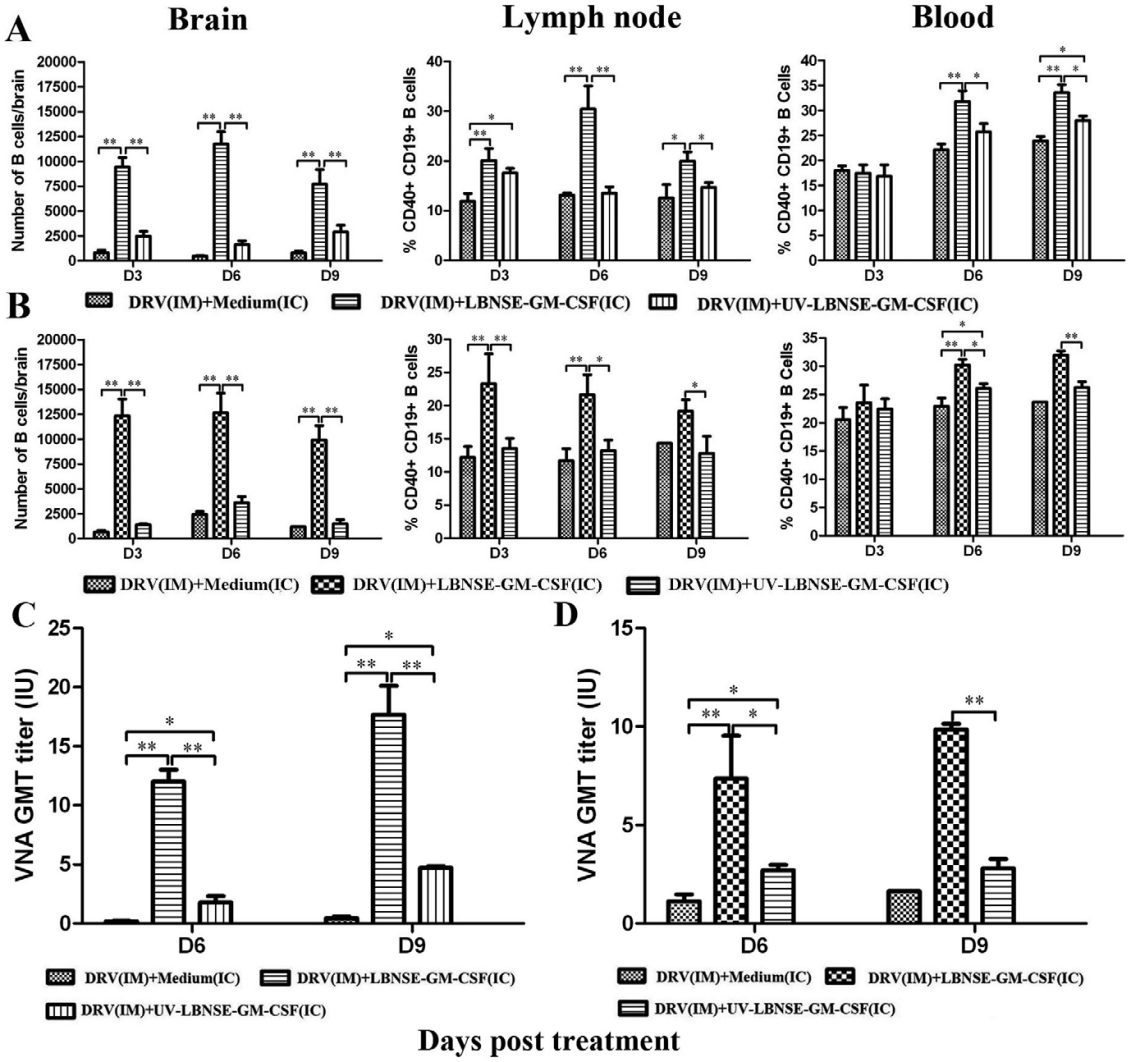
Recruitment/activation of B cells in the CNS and the periphery and the production of VNA in mice treated with LBNSE-GM-CSF. BALB/c mice were infected with 10 IMLD_50_ DRV and treated at 0 (A, C) or 4 (B, D) dpi with medium, 10^7^ FFU live or inactivated LBNSE-GM-CSF. Brain, lymph node and peripheral blood were harvested at days 3, 6, and 9 post treatments. Leukocytes recovered from CNS by Percoll centrifugation or signal-cell suspensions prepared from the lymph node or peripheral blood were stained with antibodies against B cell markers (CD40 and CD19). The stained cells were analyzed by flow cytometry. The number of B cells in the brain and the percentage of mature B cells in the lymph node or blood were presented in mice treated at 0 (A) or 4 (B) dpi. Blood samples were collected at days 6 and 9 post treatment and VNA titers determined by RFFIT (C for VNA in mice treated at 0 and D at 4 dpi). All data are from n = 3 mice in each group and presented as mean values + standard errors (SE). Asterisk indicates significant differences between the indicated experimental groups as calculated by one-way ANOVA: *, p<0.05; **, p<0.01.

Fig. 6 shows recruitment/activation of B cells in the brain, lymph nodes and blood in mice infected and treated at the same time (Fig. 6A) or in mice treated at 4 dpi (Fig. 6B). Significantly more B cells were detected during the investigation period (days 3 to 9 post treatment) in the brain and lymph node and in the blood (at days 6 and 9 post treatment) of mice treated with LBNSE-GM-CSF than sham-treated mice or mice treated with UV-inactivated LBNSE-GM-CSF. Taken together, our data indicate that ic treatment with LBNSE-GM-CSF can recruit/activate more DCs and B cells both in CNS and periphery than treatment with medium or UV-inactivated LBNSE-GM-CSF.

### Treatment with LBNSE-GM-CSF enhances the production of virus neutralizing antibodies (VNA)

To investigate if recruitment/activation of DCs and B cells lead to enhanced production of VNA, blood samples were collected from mice at days 6 and 9 post treatment and VNA determined by the RFFIT assay 20]. As shown in Fig. 6C, VNA titers were below 0.5 IU in DRV-infected and sham-treated mice at 6 and 9 dpi. In mice infected with DRV and treated with UV-inactivated LBNSE-GM-CSF, VNA titers were low (1.78 IU) at 6 but increased to 4.72 IU by 9 dpi (Fig. 6C). Mice treated with LBNSE-GM-CSF produced high level of VNA both at 6 (12.01 IU) and 9 dpi (increased to 17.67 IU) (Fig. 6C). When the treatment was initiated at 4 dpi, the VNA titers did not increase from day 6 to 9 post treatment in DRV-infected and sham-treated mice (1.13 to 1.43 IU) or in mice infected with DRV and treated with UV-inactivated LBNSE-GM-CSF (2.71 to 2.81 IU) (Fig. 6D). High VNA titers were induced in mice treated with LBNSE-GM-CSF at days 6 (7.36 IU) and 9 post treatment (increased to 8.48 IU) (Fig. 6D). These data indicate that treatment with LBNSE-GM-CSF induced significantly higher adaptive immunity as shown by VNA titers than sham-treated mice or mice treated with UV-inactivated LBNSE-GM-CSF. Nevertheless, treatment with UV-inactivated LBNSE-GM-CSF also induced relatively high level of VNA titers in the periphery.

### Enhancing the BBB permeability with MCP-1 improved the survival of mice treated with UV-inactivated LBNSE-GM-CSF

**Figure 7:**
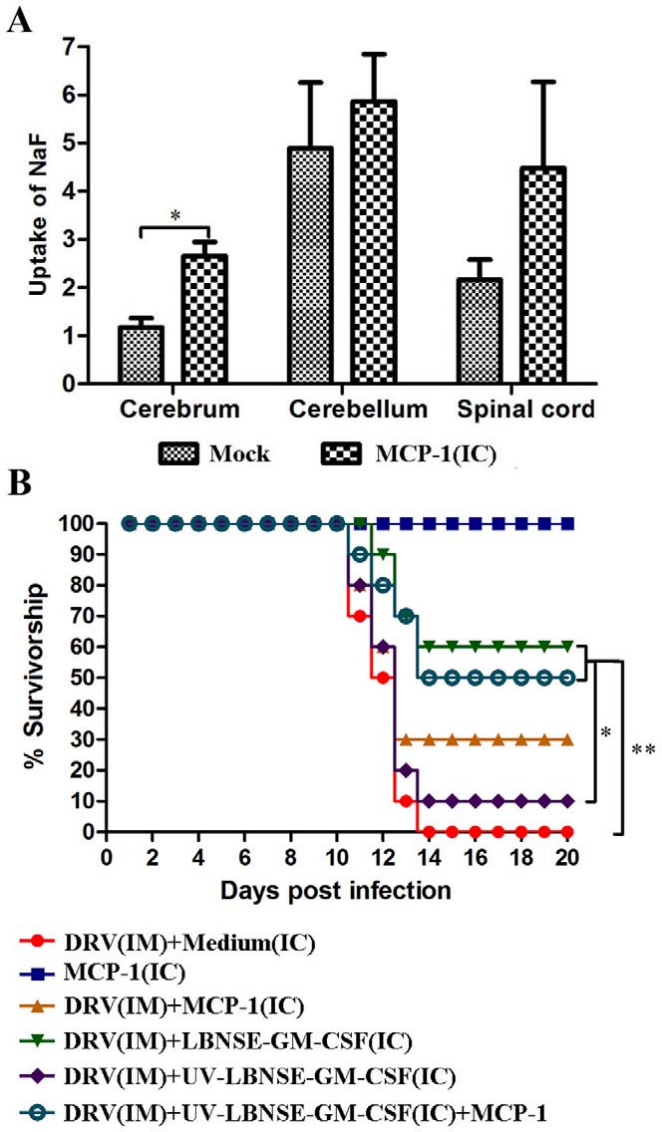
Enhancement of BBB permeability induced by MCP-1 improves protection in mice treated with UV-inactivated rRABV. Mice were treated with medium or MCP-1 (25 µg) by ic, BBB permeability was determined by uptake of sodium fluorescein at day 2 post treatment (A). ICR mice (group of 10) at age of 4–6 weeks were infected i.m. with 10 IMLD_50_ DRV, and then treated with medium, MCP-1 (25 µg), 10^7^ FFU LBNSE-GM-CSF, UV-inactivated LBNSE-GM-CSF (equivalent to 10^7^ FFU) or a mixture of MCP-1 (25 µg) and UV-inactivated LBNSE-GM-CSF (equivalent to 10^7^ FFU) by ic at day 4 post infection. The mice were observed for 20 days post infection, and survivorship was recorded and analyzed. Asterisk indicates significant differences between the indicated experimental groups as calculated by one-way ANOVA or Log-rank test: *, p<0.05; **, p<0.01.

All the above results indicate that LBNSE-GM-CSF induces the expression of innate immune molecules, infiltration of inflammatory cells into the CNS, and enhancement of BBB permeability, all of which facilitates immune effectors enter into the CNS and promotes viral clearance. To further confirm that expression of cytokines/chemokines is a direct mechanism for enhancement of BBB permeability, ICR mice were inoculated by im with 10IMLD_50_ DRV and then treated with medium, MCP-1 (25 µg), 10^7^ FFU LBNSE-GM-CSF, UV-inactivated LBNSE-GM-CSF (equivalent to 10^7^ FFU) or a mixture of MCP-1 (25 µg) and UV-inactivated LBNSE-GM-CSF by ic at 4 dpi. Mice were monitored daily for 20 days for developing diseases and deaths. As shown in Fig. 7, treatment with MCP-1 alone enhanced BBB permeability at day 2 post treatment (Fig. 7A), but did not induce any disease while 100% of the mice infected with DRV by im and treated with medium developed rabies and succumbed to the infection by 14 dpi (Fig. 7B). Treatment with MCP-1 increased the survival to 30% in mice infected with DRV, although no significant difference was found between these two groups. Treatment with MCP-1 increased significantly (*p<0.05*) the survival rate from 10% to 50% in mice infected with DRV and treated with UV-inactivated LBNSE-GM-CFS. As shown above, 60% of the mice infected with DRV and treated with LBNSE-GM-CSF survived (Fig. 7B). These data indicate that enhancement of BBB permeability allowed immune effectors induced by LBNSE-GM-CSF in the periphery infiltrate into the CNS and help clear RABV from the CNS, thereby preventing mice from developing rabies.

## Discussion

Viral infection of the CNS poses unique challenges to the immune system with regards to controlling and eliminating the invading pathogens. Traditionally, the CNS is considered an immunologically privileged site 21] and the presence of a BBB provides a physical and physiological barrier for cells and molecules to enter into the CNS 22]. Although enhancement of BBB permeability and infiltration of inflammatory cells have often been associated with pathological changes in the CNS when infected by viruses 22,23], transiently increased BBB permeability has been found to be helpful in clearing RABV from the CNS 19,24]. Induction of an autoimmune CNS inflammatory response resulted in increasing BBB permeability and aided RABV clearance 19]. Expression of triple G in SPBAAN (a recombinant RABV SAD strain) also has the ability to enhance BBB permeability and prevent infected animals from developing rabies 25]. In the present study, we showed that direct administration of chemokines or rRABV expressing GM-CSF into the CNS could help clear RABV from the CNS and prevent mice infected with street RABV from developing rabies. However, administration of rRABV via peripheral routes is not as effective as the ic route and this may be due to the quicker induction of proinflammatory mediators in the CNS by direct ic administration, which impacts the change of the BBB permeability faster than other routes of administration.

Proinflammatory mediators such as chemokines and cytokines have a prominent role in enhancement of the BBB permeability during inflammatory responses 26,27,28]. In fact, expression of chemokines early in response to infection with neurotropic viruses aids in effective host defense by promoting vascular permeability and regulating parenchymal lymphocyte infiltration 22]. In the present study, chemokine alone or cytokines expressed by rRABV are used to enhance the BBB permeability. This was based on studies by us 13,17] and others 29,30] that laboratory-adapted RABV induced high expression of innate immune genes (particularly chemokines and IFN) while street RABV did not. It is thus hypothesized that expression of chemokines and IFN is one of the mechanisms by which RABV is attenuated 13,17]. Over-expression of chemokines (MIP-1 alpha, RANTES, or IP-10) by rRABV has been found to enhance BBB permeability 14]. In the present study, it shows that over-expression of GM-CSF also enhances the BBB permeability. It is known that expression of chemokines/cytokines results in recruitment of inflammatory cells into the CNS which enhances BBB permeability 22]. Indeed direct administration of LBNSE-GM-CSF into the CNS stimulated high level expression of chemokines/cytokines (at day 3 post treatment), infiltration of inflammatory cells (neutrophils, activated microglia/macrophages and T lymphocytes at day 3–9 post treatment), and enhancement of BBB permeability (at day 6 post treatment). Direct administration of a chemokine (MCP-1) into the CNS also resulted in enhancement of BBB permeability, further supporting the role of chemokines/cytokines in the enhancement of BBB permeability.

It is apparent that enhancement of BBB permeability alone is not sufficient to prevent mice from developing rabies since administration of MCP-1 alone did not significantly increase the survival of mice infected with DRV despite the fact that BBB permeability was increased significantly in the cerebrum 2 days after administration. We believe that this might be due to the low RABV-specific immunity (VNA) in the mice infected with DRV (1.13 to 1.43 IU at 10 and 13 dpi). However, administration of MCP-1 did significantly improve protection in animals infected with DRV and treated with UV-inactivated LBNSE-GM-CSF (2.71 to 2.81 IU at 10-13 dpi). In fact the rate of protection (50%) in mice treated with MCP-1 and UV-inactivated LBNSE-GM-CSF is close to that (60%) afforded by treatment with live LBNSE-GM-CSF although the VNA titers in mice treated with the latter (about 7 IU) are much higher. The high level of VNA induced by LBNSE-GM-CSF is likely due to the activation of the innate immune responses such as expression of chemokines/cytokines and the recruitment/activation of DCs as reported previously 15,16] and demonstrated in the present study. We have consistently observed that infection with street RABV by either ic 13] or im 17,31] fails to stimulate the innate immune responses while laboratory-adapted RABV induces quick and high level of such responses including expression of innate immune molecules in and infiltration of inflammatory cells into the CNS. Infection with DRV stimulated the expression of innate immune genes only at the late stage of the infection, but did not induce infiltration of inflammatory cells. As a consequence, enhancement of BBB was only observed at 9 dpi and only in the spinal cord. In addition, the adaptive immune responses (VNA titers) in mice infected with DRV are significantly lower than those in mice infected with laboratory-adapted RABV. This is not surprising since it has been reported that only 20% rabid patients have detectable VNA at the time of death 32]. Roy et al 18] reported that infection with street RABV induced similar levels of innate and adaptive immune responses in mice as laboratory-adapted RABV. However, infection with street RABV failed to enhance the BBB permeability and thus the immune effectors could not gain entry into the CNS to clear the virus. The discrepancies between these studies could be due to differences in the virus doses used. We consistently infect animals with 10 IMLD_50_ or ICLD_50_ 13,17] and Roy et al 18] infected mice with virus titers as titrated in cell culture (FFUs). Since we have reported that street RABV is at least 1000 times more virulent than the laboratory-adapted RABV, and thus it requires more virus (FFU) for laboratory-adapted RABV to kill the infected mice 13]. Although infection with high doses of street RABV induced comparable adaptive immunity (VNA) as the laboratory-adapted RABV in the periphery, it may not have stimulated chemokine expression in time to enhance the BBB permeability. Thus, enhancement of BBB permeability and the development of RABV-specific immunity, i.e. VNA, are the two major factors contributing to the clearance of RABV from the CNS and the protection of mice from developing rabies.

Over-expression of chemokines/cytokines in RABV not only enhances the BBB permeability, but also increases the adaptive immune responses. Previously it was found that immunization with rRABV expressing MIP-1α, MDC, or GM-CSF induced significantly more VNA responses in mice, which led to better protection than with the parent virus when challenged with virulent RABV 15,16]. The increased immunogenicity is due to recruitment/activation of DCs. Indeed, direct ic administration of LBNSE-GM-CSF into the CNS recruited/activated DCs in the periphery. Not only significantly more CD11c+ cells, but also higher geometric mean fluorescence of the CD80 and CD86 expression, were detected in the lymph nodes and blood of mice treated with LBNSE-GM-CSF than in sham-treated mice or in mice treated with UV-inactivated LBNSE-GM-CSF. Recruitment/activation of DCs resulted in significantly more activation of B cells in the CNS of mice treated with LBNSE-GM-CSF than in sham-treated mice or mice treated with UV-inactivated LBNSE-GM-CSF. It has been hypothesized that it is the VNA produced in situ (CNS) by invading B cells, rather than those produced in the periphery and then transported into the CNS, that are protective 33]. In our study, direct ic administration of UV-inactivated LBNSE-GM-CSF did not provide better protection than sham-treatment despite the fact that VNA was induced in the periphery. Very few B cells were detected in the CNS. However, treatment with UV-inactivated LBNSE-GM-CSF together with administration of MCP-1 improved the rate of survival to the level similar to that in mice treated with live LBNSE-GM-CSF. It is not known whether administration of MCP-1 allowed VNA and/or B cells enter into the CNS and thus future studies are warranted.

Traditional inactivated RABV vaccines have been used for pre- and post-exposure prophylaxis for humans with high safety and efficacy 34,35,36]. However, multiple doses are needed to stimulate adequate immunity, thus driving up the cost and preventing wide distribution of these vaccines, particularly in the developing countries 2]. Furthermore, inactivated RABV vaccines cannot be used for delayed treatment since they may accelerate the development of rabies 12]. Live attenuated RABV and recombinant live vaccinia virus expressing RABV G (VRG) have been so far limited to immunization of wildlife animals 37,38,39]. Recently, it has been shown that live-attenuated RABV, particularly those expressing multiple copies of RABV G or immune stimulating molecules, can further attenuate viral virulence and at the same time enhance the immunogenicity 14,15,16,25,40], suggesting that live-attenuated RABV (expressing multiple copies of the G or immune stimulatory molecules) could be developed as RABV vaccines for pre- and post-exposure prophylaxis. Indeed, Wu et al (34) reported that co-administration of live-attenuated RABV and street RABV can protect mice and hamsters from developing rabies. Furthermore, Faber et al (24) reported that 50% of the mice survived the infection with a street RABV when given a recombinant RABV expressing three copies of the G as late as 4 dpi. In this study, significantly more mice were protected when recombinant RABV expressing GM-CSF was given as late as 5 dpi. Most importantly, these live recombinant RABV can enhance the BBB permeability, allowing innate as well as RABV-specific immunity to enter the CNS to clear RABV as shown previously (24) as well as in this present study. Therefore, these commodities may have practical implications in treatment of clinical rabies. Further studies are needed to address if such treatment is effective at the time of clinical diseases in a more suitable animal model.

## Materials and Methods

### Ethics Statement

This study was carried out in strict accordance with the recommendations in the Guide for the Care and Use of Laboratory Animals of the National Institutes of Health. All animal experiments were carried out as approved by the Institutional Animal Care and Use Committee, University of Georgia (AUP # A2009 5–103, animal welfare assurance no. A3085-01). All efforts were made to minimize animal suffering.

### Cells, viruses, antibodies, and animals

BSR cells, a cloned cell line derived from BHK-21, were maintained in Dulbecco's modified Eagle's medium (Mediatech, Herndon, VA) supplemented with 10% fetal bovine serum (FBS) (Gibco, Grand Island, NY). Mouse neuroblastoma cells (NA) were maintained in RMPI 1640 medium (Mediatech) containing 10% FBS. Recombinant RABV expressing GM-CSF (LBNSE-GM-CSF) was constructed as described 16]. Street RABV (DRV) 41] (Mexico-origin, data not published) was propagated in suckling mouse brains. Challenge virus standard (CVS)-11 was propagated in NA cells. Fluorescein isothiocyanate (FITC)-conjugated antibody against the RABV N protein was purchased from Fujirebio (Melvin, PA). Antibodies used for flow cytometric analysis, such as anti-CD3 (clone 17A2), anti-Ly6G (clone RB6-8C5), anti-CD45 (clone 30-F11), anti-CD11b (clone M1/70), anti-CD11c (clone HL3), anti-CD80 (clone 16-10A1), anti-CD86 (clone GL1) and anti-CD40 (clone 3/23) were purchased from BD Pharmingen (San Jose, CA). Anti-CD19 antibody (clone 1D3) was purchased from eBioscience (San Diego, CA). Recombinant murine MCP-1 was purchased from PreproTech (Rocky Hill, NJ). Female BALB/c and ICR mice were purchased from Harlan and housed in temperature- and light-controlled quarters in the Animal Resources Facility, College of Veterinary Medicine, University of Georgia. At indicated time points, mice were anesthetized with ketamine-xylazine at a dose of 0.1 ml/10 g body weight and then perfused by intracardiac injection of PBS followed by 10% neutral buffered formalin as described previously 42]. Brain tissues were collected for respective studies.

### Virus titration

Mouse brains were homogenized in phosphate-buffered saline (PBS) (10 ml/g tissue). After homogenization and centrifugation, the supernatants were harvested, aliquoted and stored at -80°C until use. Viruses were titrated by direct fluorescent antibody assay (dFA) in NA cells. NA cells in 96-well plate were inoculated with serial 10-fold dilutions of supernatant and incubated at 34°C for 2 days. The culture supernatant was removed and the cells were fixed with 80% ice-cold acetone for 30 min. The cells were then stained with FITC-conjugated anti-RABV N antibodies. Antigen-positive foci were counted under a fluorescent microscope (Zeiss, Germany) and viral titers were calculated as fluorescent focus units (FFU) per milliliter. All titration were carried out in quadruplicate.

### Quantitative real-time RT-PCR (qRT-PCR)

To quantify the expression of chemokines/cytokines, a real time SYBR Green PCR assay was carried out in an Mx3000P apparatus (Stratagene, La Jolla, CA). Brain tissues were removed from infected mice after perfusion at indicated time points, and flash frozen on dry ice before being stored at −80°C. Total RNA was extracted with Trizol and used for qRT-PCR as described previously 13]. Each reaction was carried out in duplicate with approximately 100 ng of DNase-treated RNA and 5 nM each primer pairs described previously 13] by using a One-Step Brilliant II SYBR green qRT-PCR master mix kit (Stratagene, La Jolla, CA) according to the manufacturer's instruction. For chemokines/cytokines expression, mRNA copy numbers of a particular gene were normalized to the housekeeping gene glyceraldehyde-3-phosphate dehydrogenase (GAPDH). Levels of gene expression in a test sample are presented as the fold increase over that detected in sham-infected controls.

### Measurement of BBB permeability

BBB permeability was measured using a modification of a previously described technique in which a low-molecular-weight fluorescent marker (Sodium fluorescein; molecular weight, 376) is utilized as a tracer molecule 24]. Briefly, mice received 100 µl of 100 mg/ml sodium fluorescein intravenously under anesthesia. After 10 min circulation for sodium fluorescein, peripheral blood was collected and mice were perfused with PBS for 10 min. Serum (50 µl) was recovered and mixed with an equal volume of 15% trichloroacetic acid (TCA). After centrifugation for 10 min at 10,000×g, the supernatant was harvested. Spinal cord or brain tissues were homogenized in 7.5% TCA and centrifuged for 10 min at 10,000×g to remove insoluble precipitates. After the addition of 30 µl 5 M NaOH to 120 µl supernatant, the fluorescence was determined using a BioTek Spectrophotometer (Bio-Tek Instruments Inc, Winooski, VT) with excitation at 485 nm and emission at 530 nm. Sodium fluorescein taken up into tissue is expressed as (µg fluorescence in cerebrum/mg tissue)/(µg fluorescence/ml sera) to normalize uptake values of the dye for blood levels of the dye at the time of tissue collection.

### Leukocyte isolation from CNS, lymph nodes, and the blood for flow cytometry

Mouse brains were harvested and digested with 1 µg/µl DNase I (Sigma-Aldrich), 2 µg/µl collagenase D (Worthington Biochemical Corporation, Lakewood, NJ) in Hanks balanced salt solution (with Ca^2+^ and Mg^2+^) for 1 h to disperse the tissue into single-cell suspension. Viable cells were separated by discontinuous Percoll gradient (70/30%) centrifugation (650×g, 25 min). After being washed once with Hanks balanced salt solution (without Ca^2+^ and Mg^2+^) and counted, cells were stained for antibodies against CD3, Ly6G, CD45, CD11b and CD19 at 4°C for 30 min and then fixed with 1% paraformaldehyde for flow cytometry.

Cervical lymph nodes and blood were collected and red blood cells were lysed by ammonium chloride lysing solution (150 mM NH_4_Cl, 10 mM NaHCO_3_, 1 mM EDTA). Single-cell suspensions were then prepared at 10^6^ cells in PBS containing 2% FBS and 0.1% NaN_3_ and stained with antibodies against CD40, CD19, CD11b, CD11c, CD80 and CD86 for 30 min at 4°C. Data were acquired on LSR-II flow cytometer (BD Bioscience), and analyzed using BD FACSDiva (BD Pharmingen) or FlowJo software (Tree Star).

### Rapid fluorescent focus inhibition test (RFFIT)

Blood was collected for measurement of VNA using the RFFIT as described previously 20]. Briefly, 50 µl of serial five-fold dilutions of serum were prepared in Lab-Tek Chamber slides (Nalge Nunc International, Rochester, NY). Fifty FFD_50_ (50% Fluorescing Foci dose) of CVS-11 was added to each chamber and incubated for 90 min at 37°C. NA cells (10^5^ cells) were added into each chamber and the slides were incubated at 37°C for 20 hr. Then the cells were fixed with ice-cold 80% acetone and stained with FITC-conjugated anti-RABV N antibodies for 1 hr at 37°C. Twenty fields in each chamber were observed under a fluorescent microscope, and the 50% endpoint titers were calculated according to the Reed-Meunch formula 43]. The values were compared with that of the reference serum (obtained from the National Institute for Biological Standards and Control, Herts, UK) and normalized to international units (IU/ml).

#### Statistical Analyses

Statistical significance of the differences between groups was determined using one-way ANOVA or Log-rank test.
